# Teledermatology: The Most Recent Advancements and Applications of Mobile Apps: A Narrative Review

**DOI:** 10.1002/hsr2.72521

**Published:** 2026-05-19

**Authors:** Fateme Molaalinejad, Farideh Eghdampour, Lida Menati, Kowsar Qaderi, Ahmadreza Shamsabadi

**Affiliations:** ^1^ Health Information Technology, Department of Health Information Management, School of Allied Medical Sciences Tehran University of Medical Sciences Tehran Iran; ^2^ Department of Midwifery Islamic Azad University Marand Iran; ^3^ Midwifery Department, School of Nursing and Midwifery Kermanshah University of Medical Sciences Kermanshah Iran; ^4^ Fertility and Infertility Research Center, Health Technology Research Institute Kermanshah University of Medical Sciences Kermanshah Iran; ^5^ Health Information Management, School of Medical Sciences, Esfarayen Faculty of Medical Sciences Esfarayen Iran

**Keywords:** applications, dermatological, mobile health, review, skin, teledermatology, telemedicine

## Abstract

**Background and Aims:**

Mobile health improves patient–provider communication and expands access to specialists, especially in underserved areas. Training and empowerment of professionals and patients are among key objectives of mHealth technologies. This study investigated the most recent advancements and applications of mobile apps in dermatology.

**Methods:**

We conducted a review of original articles on advancements and applications of mobile apps in dermatology published from 2015 to 2024. Searches spanned Web of Science, Scopus, and PubMed using relevant keywords. Two independent authors screened and selected studies based on the inclusion criteria.

**Results:**

The most frequently cited technologies were mobile health, mobile apps, telemedicine, teledermoscopy, artificial intelligence, image processing, deep learning, clinical image analysis, and cloud‐based data storage. The primary devices were tablets or smartphones. Image optimization utilized high‐performance GPUs—notably the NVIDIA Tesla P4—followed by intensive image processing with neural network algorithms and deep learning libraries such as *timm* and *torchvision* for image analysis and processing. Notable applications included teledermoscopy applications, Guaral‐1ST, eSkinHealth, and NLR SkinApp. Teledermatology services extend beyond the capture and transmission of skin images for remote triage or consultation. Clinical decision support, referral pathways, and treatment monitoring and management—including tracking therapeutic responses and documenting short‐ and long‐term adverse effects—have also emerged as highly utilized features.

**Conclusion:**

Teledermatology can strengthen frontline healthcare capacity by improving access to resources in underserved settings. Additional applications include statistical reporting and analysis, data storage within electronic health records (EHRs), early detection and prevention of skin cancer, virtual and online consultations, and protection of patient information using barcodes. These benefits are particularly relevant in rural and remote tropical regions where skin diseases are prevalent. Offline‐capable systems are especially valuable in contexts with limited internet connectivity.

## Summary

- Mobile health (mHealth) applications are increasingly used for self‐management, early diagnosis, and screening of skin conditions, especially in underserved areas.
- AI‐powered tools within these apps enhance diagnostic accuracy and speed, supporting both patients and healthcare professionals.
- Education and empowerment are central goals—mHealth platforms help train users and improve communication between patients and dermatologists.
- Telemedicine reduces in‐person visits and healthcare workload, but challenges remain, including privacy concerns and internet accessibility.
- Offline functionality and standardized interfaces improve usability and broaden access to dermatological care.

## 1 Introduction

The skin is the largest organ of the human body, comprising the epidermis, dermis, and specialized structures such as hair, nails, and sebaceous and sweat glands [1]. This remarkable organ plays a crucial role in physiological protection, serving as a chemical and physical barrier against external factors including pathogens, ultraviolet radiation, chemicals, temperature variations, and dehydration [2, 3]. Consequently, skin care extends beyond mere surface maintenance and constitutes an essential component of overall health [4].

Engagement with skin diseases can have diverse and serious implications, including reduced quality of life, increased risk of infection transmission, and psychological‐social challenges [5]. According to global statistics, dermatological conditions represent the fourth most common group of diseases worldwide, affecting nearly one‐third of the global population [6]. Preventive strategies and contemporary knowledge in the field of skin care have enabled the mitigation of various skin health issues [1]. Furthermore, in cases of disease, the adoption of innovative medical approaches and targeted therapies can facilitate recovery and accelerate skin regeneration [6].

With the rapid advancement of information technology, healthcare providers and caregivers are increasingly adopting telemedicine and remote healthcare solutions, recognizing their significant potential to enhance patient management and treatment outcomes [7, 8]. Over the past decade, the global prevalence of smartphone usage has grown consistently. By the end of the second quarter of 2019, approximately 40,596 medical‐related applications were available on the Google Play Store, and 47,878 applications were listed on the Apple App Store [9]. Data indicate a concomitant increase in dermatology‐specific applications, particularly those focused on self‐care, aligning with this expanding trend in mobile health (mHealth) app development [10]. Awareness and familiarity among dermatologists regarding these applications play a crucial role in their effective integration into clinical practice [11].

Telemedicine is gradually transforming healthcare delivery by providing greater accessibility to specialists, reducing healthcare costs, and improving the overall quality of care [12]. The concept of mHealth encompasses medical, therapeutic, and public health services delivered via mobile devices, facilitating the provision of healthcare support across diverse settings [11]. An extensive array of smartphone applications exists that can be utilized to optimize performance across clinical, academic, and research domains. Among the most prevalent applications of mHealth are text messaging services and mobile applications, which can be effectively utilized within teledermatology [13]. Teledermatology, as a specialized branch of telemedicine, has extended skin healthcare services beyond traditional clinical settings, enabling remote consultation and management [14]. Over the past decade, the routine deployment of mHealth services in dermatology has seen a marked increase, becoming an integral part of everyday practice [15].

Moghadasi and colleagues conducted a study examining the role of mHealth in dermatological conditions and found that mHealth has significant potential to deliver diagnostic and therapeutic services, particularly in underserved regions [16]. Hadeler and colleagues also investigated the prospects and future development of mobile application programs in dermatology, concluding that the expansion of such applications is ongoing and could represent a pivotal advancement in the management of dermatological conditions [17].

The development of skin‐related applications is advancing rapidly. In recent years, there has been no comprehensive study focusing on the evaluation of mHealth applications for skin self‐care. Therefore, this review aims to assess the role of mHealth and mobile applications in the domain of skin self‐care.

## 2 Methods

This study is a narrative review that examines original articles published between 2015 and 2024 related to self‐management of dermatological conditions. Articles were retrieved through comprehensive searches in the Web of Science, Scopus, and PubMed databases, utilizing the following search strategy for the period from 2015 to December 7, 2024.

Skin OR keratinocyte OR derm OR epiderm OR cutaneous OR dermatology OR “Integumentary System” AND m‐health OR Mhealth OR “Mobile Health” OR telemedicine OR e‐health OR tele‐monitoring OR telehealth OR telecare OR teleconsultation OR “Teledermatology.”

Articles were independently reviewed by two investigators based on predefined inclusion criteria. Discrepancies were resolved through discussion with a third reviewer to determine eligibility for inclusion.

**Inclusion criteria:**

- Articles published in English.
- Original research articles focusing on the role of mHealth and mobile applications in skin self‐care.
- Publications from 2015 to 2024.

**Exclusion criteria:**

- Meta‐analyses, reviews, and nonoriginal articles.
- Conference abstracts and manuscripts without full‐text availability.
- Studies with inconclusive or nondefinitive results.
- Ongoing clinical trials without published outcomes.

### 2.1 Data Extraction

The included articles were thoroughly reviewed by two members of the research team. Based on the study objectives, a data extraction table was constructed, capturing the following information: primary author, publication year, country, study objectives, utilized infrastructure, capability, applications, and technology type.

Because this is a review of published literature and does not involve human participants, primary data collection, or any intervention, ethical approval and informed consent are not required.

## 3 Results

A total of 181 articles were retrieved from database searches: PubMed (69), Scopus (78), ISI (37), and other sources (13). After removing duplicates, 117 articles remained. Of these, 74 articles did not meet the inclusion criteria and were excluded, leaving 8 articles for final review. Data extracted from the selected articles are presented in Table 1.

**Table 1:** Characteristics of studies included in the systematic review.

ID	Year	First author	Country	Goal	Infrastructure	Capability	Application	Technology type
1 [18]	2023	Debarpan Das	Canada	System development based on artificial intelligence Direction Diagnosis of skin moles in images and initial triage and screening patients	Tablet/Smartphone NVIDIA Tesla P4 graphics card Image processing tools (Deep learning libraries (Like timm and torchvision)) Optimization techniques (Includes stochastic gradient descent (SGD)) Neural network algorithms (Like NesT)	Diagnosis Skin moles Available in pictures with 93.4% accuracy Simple and efficient structure compared to similar models Usability in triage environments Ability to train AI models with larger data sets and increase system accuracy	Reducing patient waiting time to see specialists, providing basic information to doctors and patients about the patient's skin condition, prioritizing cases that require in‐person examination	Telemedicine and mhealth, Artificial intelligence with a focus on deep learning
2 [19]	2020	Monika Janda	Australia	Taking advantage of Health Technology Companion (Healthm) and social media Direction Prevention and early detection of skin cancer	Tablet/Smartphone (With dermatoscope capability.) Tele applications Dermoscopy (Possibility of skin image analysis) Social media	Self‐management Skin lesions by patients. Ability to send images Skin To doctors for Triage and assessment. Increasing patients' access to specialized services and reducing the need for in‐person visits.	Early detection of skin cancer Education and Raising awareness Direction Prevention Diseases and Cancer Skin conditions	Telemedicine and mhealth Mobile applications Trap Dermoscopy Technologies Skin image analysis
3 [20]	2023	Alexandra Cossio	Colombia	Investigating the effectiveness of a strategy based on mHealth to monitor treatment of patients with cutaneous leishmaniasis in rural communities and compare it with standard care	Tablet/Smartphone Guaral1ST mobile application (To monitor treatment, drug side effects, and assess treatment response.) Cloud Storage/Cloud Computing	Ability to record demographic information, location and number of lesions, drug side effects, and treatment response of patients. Sending pictures of skin lesions to the doctor	Facilitate treatment follow‐up Better access to health services for patients in remote areas Checking the effectiveness of treatment	Telemedicine and mhealth Statistical software such as Stata and R for quantitative data analysis
4 [21]	2021	Rowan W. Sanderson	Australia	Presenting the first application of mobile phone camera‐based optical elastography (SBOP) technology for the assessment and diagnosis of burn scars with emphasis on use in telemedicine services	Tablet/Smartphone Imaging tools (External macro lens with 10x magnification.) Ring light LED Tools Image processing Optimization techniques	Low‐cost and lightweight design, suitable for general use. Capable of imaging with sub‐millimeter resolution (about 430 micrometers). Measurement and mapping of textures Reducing image noise using digital processing Image processing Production of mechanical stress maps	Mechanical and visual assessment of burn scars Early detection of scar areas Potential use for investigating other skin diseases or mechanical evaluations of tissues	Mhealth and Telemedicine Optical elastography
5 [22]	2022	Britney N. Wilson	America	Providing practical guidance for dermatologists to effectively and accurately assess alopecia.	Tablet/Smartphone Platform HiVirtual consultation and online consultation. Imaging tools Ring light LED	Ability to record high‐quality images from different areas of the head. Presentation Detailed instructions for preparation before the consultation Internet connection stability tests (to ensure video quality)	Assessment and diagnosis Remote alopecia Reduction The need for patients to come in person for initial consultation Providing treatment recommendations based on virtual diagnosis	Mhealth and telemedicine
6 [23]	2023	Rie R. Yotsu	America	Evaluating the usability and effectiveness of a mobile application called eSkinHealth for diagnosing and managing skin diseases in rural areas	Application eSkinHealth Amazon S3 Storage Service	Providing portable electronic medical records. A platform for teledermatology (remote consultation). Longitudinal storage and organization of patient data and clinical images. Protecting patient information using Barcode The app works offline and synchronizes data when connected to the Internet	Diagnosis and treatment of skin diseases Improving the quality of diagnosis and treatment in resource‐limited settings Increasing the capacity of the local workforce	Mhealth and telemedicine Artificial intelligence for data analysis Mobile health technology
7 [24]	2020	Kartik Dhaduk	America	Evaluation of the use of Telemedicine for skin conditions, focusing on improving work processes and increasing the accuracy of diagnosis and treatment.	Telemedicine Carts HIPAA compliant telemedicine platform Virtual consulting platforms and online consulting	Possibility of live and remote consultation between the dermatologist and the patient's primary care team. Possibility of recording consultation tips in the patient's electronic record. Providing combined consultation including clinical examination and treatment prescription.	Providing services Teledermatology, to patients admitted to hospitals Improving diagnostic accuracy for skin diseases Increasing the quality of treatment management Patients Hospitalization	Mhealth and telemedicine Tools Electronic health.
8 [25]	2024	Nelly Mwageni	Tanzania	Review The supporting role of the NLR SkinApp mobile application in increasing the accuracy of diagnosing and managing skin diseases by healthcare workers	Tablet/Smartphone Application NLR SkinApp	Coverage of 29 skin diseases Presentation Treatment suggestions and patient referrals to the experts Usable by users with limited knowledge about skin issues. Offline usability	Help To Diagnosis and management of skin diseases. Providing tools Support Decision‐making inSpecial conditions and limited resources Increasing health service coverage in rural areas	Mhealth and telemedicine Tools Support Digital decision‐making

This study is a review of eight articles published between 2020 and 2024, all of which focused on the use of mHealth technologies and telemedicine in the self‐care and self‐management of skin diseases. mHealth technology has enhanced patients' access to specialized resources and services, and has facilitated processes such as triage, diagnosis, follow‐up, and treatment management. However, most studies provide limited evidence regarding long‐term clinical effectiveness, comparisons with gold‐standard methods, and comprehensive evaluations of data security and regulatory compliance. Moreover, with the increasing integration of artificial intelligence (AI) into mHealth, the effectiveness of these approaches can be further improved.

The most commonly utilized technologies referenced across the studies included telemedicine, mHealth, and mobile applications, all of which were employed in the reviewed articles. Image processing, teledermoscopy, and clinical image analysis technologies were also mentioned in five of the studies. Additional technologies cited included AI and deep learning algorithms, as well as cloud‐based data storage solutions. Most studies on mHealth in dermatological self‐management have been conducted across five countries: the United States (three studies), Australia (two studies), and one study each from Canada, Colombia, and Tanzania.

**Figure 1:**
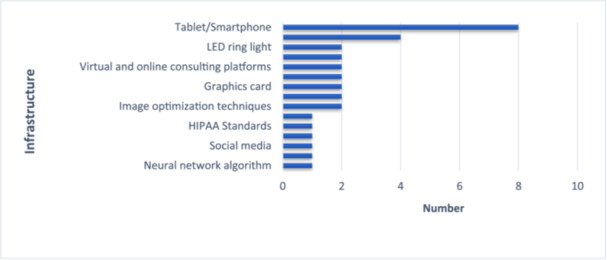
Frequency distribution of technology infrastructure in studies.

Review of the articles revealed that the technological infrastructures used in this field can be broadly categorized into several main groups, as illustrated in Figure 1. The most common infrastructure was the use of tablets or smartphones, reported in all included studies. Furthermore, depending on the objectives of each study, a variety of specialized applications were utilized, including teledermoscopy applications, the Guaral1ST app, the eSkinHealth app, and the NLR SkinApp. Each of these was selected based on the clinical needs and operational conditions of the respective study.

**Figure 2:**
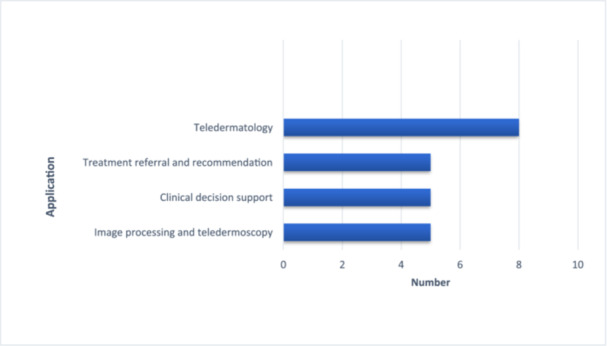
The most common uses of mobile health in skin diseases.

Analysis of the included studies revealed several key applications of mHealth technologies in dermatology. A prominent and explicit use across all eight articles was the provision of teledermatology services, involving the capture and transmission of skin images for remote triage or consultation. Image analysis and teledermoscopy—encompassing image processing and related functionalities—were highlighted in five studies [7, 18, 19, 21, 23]. Clinical decision support, referral systems, and treatment recommendations also emerged as highly utilized features, emphasized in five articles. Three studies focused on improving access to resources in underserved settings and strengthening frontline healthcare capacity. Additionally, treatment monitoring and management—including tracking therapeutic responses and documenting short‐ or long‐term adverse effects—were directly addressed in two studies and indirectly referenced in others (Figure 2—Most frequent applications of mobile health in skin disease management) [18, 21].

Other applications also include skin cancer prevention and awareness through social media, management and diagnosis of leishmaniasis, protection of patient information using barcodes, and statistical reporting/analysis for effectiveness evaluation. In two studies, image optimization techniques were employed, followed by intensive image processing tasks. This process utilized high‐performance graphics cards—particularly the NVIDIA Tesla P4—and deep learning libraries such as *timm* and *torchvision* for image analysis and processing [18, 21]. Neural network algorithms, including *NesT*, played a key role in enhancing the performance of image processing models. Data storage was also emphasized in both studies, with Cloud Storage/Cloud Computing services and the Amazon S3 Storage Service used for this purpose [20, 23].

Virtual and online consultation platforms were identified in two studies as communication channels between patients and physicians [13, 22]. One study also utilized social media to facilitate interaction and engagement between healthcare providers and patients [19]. Additional reported infrastructures included imaging tools and LED ring lights (in two studies), which contributed to improved image quality [21, 22]. Furthermore, the use of HIPAA‐compliant telemedicine platforms—specifically in the form of Telemedicine Carts—was highlighted in one study as a key infrastructure for enabling secure communication between physicians and patients across long geographic distances [13].

Another study from the United States aimed to develop a system utilizing AI tools for early diagnosis and management of chronic skin conditions, especially beneficial in rural and remote tropical regions where skin diseases are common. Notably, this system has offline capabilities, making it valuable in situations where internet access is limited [17]. In Tanzania, a mobile application was evaluated for improving the accuracy of initial diagnoses by healthcare workers; its offline functionality was highlighted as a key feature. Furthermore, the app included clinical decision support (CDS) tools, enabling users with limited medical knowledge to make more accurate and reliable decisions with guidance from the system [25].

In Australia, research focused on leveraging mHealth and social networks for skin disease self‐management, emphasizing early detection and prevention of skin cancer, including the use of teledermoscopy. Another study conducted in Australia in 2021 showcased the first application of a camera‐based optical elastography technology to assess and diagnose burn scars [19, 21].

In 2020, a US‐based study concentrated on improving workflow and enhancing diagnostic and treatment accuracy, underscoring the importance of mHealth in dermatological care for increasing precision in diagnosis [24].

Education and empowerment of dermatologists and patients constitute additional objectives of mHealth applications, which have received considerable attention in various studies [20, 22]. The use of social networks alongside mHealth has also contributed to the increased efficiency of telehealth systems for training healthcare professionals and patients [20].

One of the key missions of mHealth is to enhance the capacity of both clinicians and patients in managing and self‐managing skin diseases, particularly chronic conditions. This aim was explicitly addressed in three of the reviewed studies and implicitly supported by others [18, 20, 23].

Reviewing the reported capabilities across the included studies revealed several core functionalities. All studies emphasized the ability to capture and transmit high‐quality clinical images for evaluation, triage, treatment management, and follow‐up as a central feature. Five studies specifically utilized clinical decision support functionalities, including treatment recommendations, patient referral, and prioritization. Offline functionality and synchronization upon internet reconnection were reported in two studies. The same number of studies also highlighted data storage and organization within electronic health records (EHRs). Additionally, two studies addressed the documentation of clinical details and treatment monitoring. More specialized capabilities—such as mechanical tissue assessment, data protection via barcode systems, and provision of detailed user instructions—were reported in a limited number of studies.

## 4 Discussion

The present review highlights the broad benefits of mHealth applications in teledermatology. Most reviewed studies underscore the role of mHealth as a facilitator in enhancing communication between patients and healthcare providers and in increasing patient access to specialists and practitioners, particularly in underserved regions [23]. Additionally, the reduction in in‐person visits to clinics and healthcare facilities, leading to decreased workload for healthcare providers and increased capacity of local health personnel, alongside improvements in care quality, constitutes significant advantages of mHealth in dermatological conditions Importantly, these findings align with broader digital health literature suggesting that telemedicine interventions are most impactful in geographically dispersed or resource‐limited contexts, where specialist access is structurally constrained [24].

However, it should be noted that the majority of included studies were pilot or implementation‐focused investigations with limited sample sizes, which may restrict the generalizability of these reported benefits. The predominance of feasibility and proof‐of‐concept studies indicates that teledermatology remains in a translational phase, moving from technological innovation toward evidence‐based clinical integration.

Furthermore, the use of this technology enables virtual diagnostic and advisory services, which can expedite prevention and treatment processes while also enhancing the accuracy of diagnoses [8]. mHealth applications have also proven highly effective in educating healthcare professionals and patients, as well as in the management of chronic skin diseases [22]. Given the prevalence and nature of skin conditions, the integration of mHealth in this field has positively impacted prevention, self‐management, and treatment outcomes [19]. Nevertheless, the heterogeneity observed across study designs, outcome metrics, and technological architectures limits the ability to derive standardized performance benchmarks or pooled effectiveness estimates.

This variability also reflects the absence of universally accepted evaluation frameworks for digital dermatology tools, underscoring the need for consensus‐driven reporting standards.

Key infrastructure components for leveraging mHealth in dermatological self‐management include smartphones, computer systems with advanced graphics capabilities, internet connectivity, telemedicine platforms, data storage and processing systems, and appropriate optical imaging equipment [19, 21, 23]. AI has demonstrated considerable efficiency in data analysis—such as image analysis, natural language processing, and big data analytics—and, with increasing capabilities and accuracy, has garnered attention as a facilitating technology in the prevention, diagnosis, treatment, and self‐management of skin diseases [19, 20]. In Canada, an AI‐based system was developed to examine and screen skin lesions, particularly moles, with an emphasis on increasing diagnostic accuracy and speed through deep learning techniques [19].

In the context of AI‐assisted mHealth, techniques such as image enhancement, deep learning library optimization, and artificial neural networks are particularly valuable and effective [20, 24]. While diagnostic accuracies exceeding 90% were reported in some studies, these figures should be interpreted cautiously, as many algorithms were validated under controlled conditions and may not reflect real‐world performance across diverse skin types, lighting conditions, and imaging qualities.

The underrepresentation of darker skin phototypes in AI training data sets remains a critical equity concern that future research must address.

Studies indicate that one of the motivating factors for adopting mHealth applications for skin self‐management is having a user‐friendly, standardized interface that allows users with varying levels of knowledge to utilize the system easily and benefit from its features [18]. Thus, simplicity and ease of use are crucial for maximizing the effectiveness of these applications. Additionally, the high diagnostic accuracy of mHealth applications is a significant advantage that encourages clinicians to adopt these tools. Features supporting continuous treatment and self‐management of skin conditions are also considered essential; for example, EHRs and the ability to document consultations within patients' digital files are measures designed to facilitate this goal [20].

However, integration with existing health information systems and interoperability with national EHR infrastructures remain insufficiently examined in the current literature. Without seamless interoperability, the scalability and sustainability of teledermatology platforms may be significantly compromised.

One of the most prominent challenges in implementing this technology is safeguarding patient data privacy and security, which has been addressed in multiple studies through various protective measures [20, 21]. Another challenge pertains to ensuring access to stable internet connections for effective communication between healthcare providers and patients. Some applications incorporate offline functionalities, allowing data synchronization once internet access is restored, or utilize network testing tools to verify connection quality [20, 25]. Beyond technical barriers, medico‐legal accountability, cross‐jurisdictional licensing, and reimbursement policies represent structural determinants that influence long‐term adoption of teledermatology services.

These regulatory and financial dimensions are often overlooked in technological evaluations but are central to real‐world implementation.

This review has several limitations including: discussed methodological heterogeneity, small evidence base, and lack of long‑term data. Only eight studies met the inclusion criteria, which may limit the comprehensiveness of the findings. The strict inclusion criteria and focus on recent publications may have excluded earlier foundational or exploratory studies that contributed to technological innovation in teledermatology. Because this study is a narrative review, formal risk‑of‑bias assessment is not mandatory. No formal risk‐of‐bias assessment was conducted, and therefore the strength of the summarized evidence should be interpreted with caution. Nevertheless, the heterogeneity observed across study designs, outcome metrics, and technological architectures limits the ability to derive standardized performance benchmarks or pooled effectiveness estimates.

Additionally, publication bias may have favored studies reporting positive technological outcomes, potentially overestimating overall effectiveness.

Future research should move beyond diagnostic accuracy indicators and consider patient‐reported outcomes, long‐term adherence data, health economic analyses, and implementation science frameworks. Large‐scale pragmatic trials and multicenter collaborations will be essential to determine the sustained impact of these technologies at the population level.

## 5 Conclusion

The application of mHealth across various medical fields, including dermatology, creates extensive opportunities for disease prevention, treatment, and self‐management. Given the significant role of self‐management in overall patient health, the deployment of mHealth solutions has been implemented in multiple countries, and with increasing awareness of skin‐related issues and improved access to resources and specialists—especially in developing regions—these tools not only enhance patients' quality of life but also improve overall patient satisfaction with care. mHealth can contribute to increased treatment efficacy, system efficiency, and reduction of healthcare provider workload.

Despite these promising developments, teledermatology should currently be viewed as a complementary extension of conventional dermatological services rather than a complete substitute. Its long‐term success will depend on rigorous clinical validation, equitable algorithm development, regulatory harmonization, economic sustainability, and integration into routine care pathways.

Strategic alignment between technological innovation, clinical governance, and public health priorities will be critical to unlocking the full transformative potential of mobile dermatology platforms in the coming decade.

## Author Contributions

**Fateme Molaalinejad:** methodology, investigation, writing – original draft, writing – review and editing, visualization, conceptualization. **Farideh Eghdampour:** writing – original draft, writing – review and editing, validation, data curation. **Lida Menati:** writing – review and editing, writing – original draft, data curation, visualization‘ validation. **Kowsar Qaderi:** investigation, supervision, methodology, writing – review and editing, writing – original draft. **Ahmadreza Shamsabadi:** investigation, validation, writing – review and editing, formal analysis, project administration, supervision, software, methodology, conceptualization. All authors have read and approved the final version of the manuscript.

## Funding

The authors have nothing to report.

## Ethics Statement

Ethics declaration: IR.ESFARAYENUMS.REC.1403.013. This article is based on published data, and hence no ethical approval is required.

## Consent

As this is a review of published literature and does not involve human participants, primary data collection, or any intervention, informed consent is not required.

## Conflicts of Interest

The authors declare no conflicts of interest.

## Transparency Statement

The lead authors Kowsar Qaderi and Ahmadreza Shamsabadi affirm that this manuscript is an honest, accurate, and transparent account of the study being reported; that no important aspects of the study have been omitted; and that any discrepancies from the study as planned (and, if relevant, registered) have been explained.

## Data Availability

Data sharing is not applicable to this article as no data sets were generated or analyzed during the current study. Kowsar Qaderi and Ahmadreza Shamsabadi had full access to all of the data in this study and take complete responsibility for the integrity of the data and the accuracy of the data analysis.
